# Characterisation of the paralytic shellfish toxin biosynthesis gene clusters in *Anabaena circinalis *AWQC131C and *Aphanizomenon sp*. NH-5

**DOI:** 10.1186/1471-2091-10-8

**Published:** 2009-03-30

**Authors:** Troco K Mihali, Ralf Kellmann, Brett A Neilan

**Affiliations:** 1 School of Biotechnology and Biomolecular Sciences, The University of New South Wales, Sydney, NSW 2052, Australia; 2 Department of Molecular Biology, University of Bergen, P.O. Box 7803, 5020 Bergen, Norway

## Abstract

**Background:**

Saxitoxin and its analogues collectively known as the paralytic shellfish toxins (PSTs) are neurotoxic alkaloids and are the cause of the syndrome named paralytic shellfish poisoning. PSTs are produced by a unique biosynthetic pathway, which involves reactions that are rare in microbial metabolic pathways. Nevertheless, distantly related organisms such as dinoflagellates and cyanobacteria appear to produce these toxins using the same pathway. Hypothesised explanations for such an unusual phylogenetic distribution of this shared uncommon metabolic pathway, include a polyphyletic origin, an involvement of symbiotic bacteria, and horizontal gene transfer.

**Results:**

We describe the identification, annotation and bioinformatic characterisation of the putative paralytic shellfish toxin biosynthesis clusters in an Australian isolate of *Anabaena circinalis *and an American isolate of *Aphanizomenon sp*., both members of the *Nostocales*. These putative PST gene clusters span approximately 28 kb and contain genes coding for the biosynthesis and export of the toxin. A putative insertion/excision site in the Australian *Anabaena circinalis *AWQC131C was identified, and the organization and evolution of the gene clusters are discussed. A biosynthetic pathway leading to the formation of saxitoxin and its analogues in these organisms is proposed.

**Conclusion:**

The PST biosynthesis gene cluster presents a mosaic structure, whereby genes have apparently transposed in segments of varying size, resulting in different gene arrangements in all three *sxt *clusters sequenced so far. The gene cluster organizational structure and sequence similarity seems to reflect the phylogeny of the producer organisms, indicating that the gene clusters have an ancient origin, or that their lateral transfer was also an ancient event. The knowledge we gain from the characterisation of the PST biosynthesis gene clusters, including the identity and sequence of the genes involved in the biosynthesis, may also afford the identification of these gene clusters in dinoflagellates, the cause of human mortalities and significant financial loss to the tourism and shellfish industries.

## Background

Paralytic shellfish poisoning (PSP) is a syndrome acquired through the consumption of contaminated shellfish or drinking water. Its symptoms include numbness and ascending paralysis followed by respiratory arrest [1]. Toxicity is mediated by a group of toxins collectively referred to as paralytic shellfish toxins (PSTs) or saxitoxins (STX).

The global occurrence of PSTs coupled with their chemical stability and high toxicity, presents a formidable problem for marine and freshwater regulating bodies, while detrimentally affecting the health of humans and animal worldwide [2,3]. STX and its analogues are potent neurotoxic alkaloids. PSTs have been shown to specifically block voltage-gated sodium and calcium channels [4,5], and prolong the gating of potassium channels in heart cells [6]. This mechanism prevents the conduction of a neural action potentials, paralysing the victim, and has also been shown to exhibit a cardio-depressory effect [1]. Additionally, the globally observed abundance of PST producing microorganisms, specifically dinoflagellates, cause substantial economic damage to the fishing industry, mainly due to closure of fisheries affected by PST producing blooms, as well as the regulatory requirement for expensive toxin monitoring programs [7].

**Figure 1:**
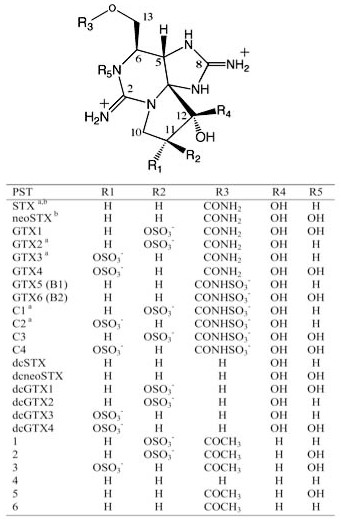
**Chemical structure of the major paralytic shellfish toxins (PST)**. ^a ^PSTs identified in *Anabaena circinalis *AWQC131C, ^b ^PSTs identified in *Aphanizomenon. sp*. NH-5. STX, saxitoxin; GTX, gonyautoxin; dc, decarbamoyl.

The parent compound of PSTs, STX, is a tricyclic perhydropurine alkaloid, which can be substituted at various positions, leading to more than 30 naturally occurring STX analogues [8-13] (Figure 1). The synthesis of PSTs has been reported in freshwater and marine organisms alike, spaning two kingdoms of life. The marine microorganisms, dinoflagellates, belonging to the genera *Alexandrium*, *Pyrodinium *and *Gymnodinium *have been reported to produce PSTs [14-16]. In freshwater systems several filamentous species of cyanobacteria, such as *Anabaena*, *Aphanizomenon*, *Cylindrospermopsis *and *Lyngbya *are also known to produce PSTs [17-20].

The occurrence of a neurotoxin, originally termed aphatoxin, from *Aphanizomenon flos-aquae *was first demonstrated by Sawyer et al. [21], where a copper sulfate treatment of a cyanobacterial bloom led to the death of six tonnes of fish in Kezar Lake, New Hampshire. PST production was later confirmed in the cultured strain *Aphanizomenon flos-aquae *NH-5, isolated from a small pond near Durham, New Hampshire. This study identified the presence of saxitoxin (STX) and neosaxitoxin (neoSTX), together with further unidentified toxic fractions [17]. Li et al. [22] re-evaluated the morphological based taxonomy of the toxic *Aphanizomenon flos-aquae *NH-5 strain. They compared the 16S rRNA gene sequences and morphology of this strain with six other strains of *Aphanizomenon flos-aquae *and reclassified it as *Aphanizomenon sp*. NH-5. According to morphological and gene sequence data, four of the investigated strains of *Aphanizomenon flos-aquae*, that have not been shown to produce toxins, are grouped together in the phylogenetic tree and were delineated from the branch represented by the two known toxin-producing *Aphanizomenon *isolates. Furthermore, Li et al. [23] reclassified the paralytic shellfish toxin-producing *Aphanizomenon flos-aquae *LMECYA 31 as *Aphanizomenon issatschenkoi *based on morphological and 16S rRNA gene sequence characteristics. The *Aphanizomenon *strains in this study formed a monophyletic cluster with three other species designations; the fascicle-forming *Aphanizomenon flos-aquae*, *Aphanizomenon gracile *strains having solitary trichomes, and *Aphanizomenon issatschenkoi *strains characterized by solitary trichomes that have tapered ends. More recently, a different *Aph. flos-aquae *strain from Portugal has been isolated and shown to produce the PSTs, STX, neosaxitoxin (neoSTX), gonyautoxin 5 (GTX5), gonyautoxin 6 (GTX6) and decarbamoyl saxitoxin (dcSTX) [3].

Similarly, *Anabaena circinalis *is a common toxic bloom-forming planktonic freshwater cyanobacterium with a global distribution and an unusual geographical segregation of toxin production [24]. Australian reports of neurotoxic cyanobacterial blooms have principally implicated *A. circinalis *as the toxin producing species. In 1991 a neurotoxic bloom of *A. circinalis *covering over 1000 km of the Darling River in Australia, reportedly with concentrations of up to 500,000 cells per millilitre, was the cause of cattle mortality [25,26]. A following comprehensive study of the Murray-Darling basin in 1994 implicated *A. circinalis *in all neurotoxic blooms in Australia, and also showed *A. circinalis *to produce PSTs, mainly STX, GTX2/3 and dcGTX2/3 [18,27]. A detailed chemical analysis of the cultured strain *A. circinalis *AWQC131C revealed the presence of STX, GTX2/3, C1/2, dcSTX and dcGTX2/3 [28].

**Figure 2:**
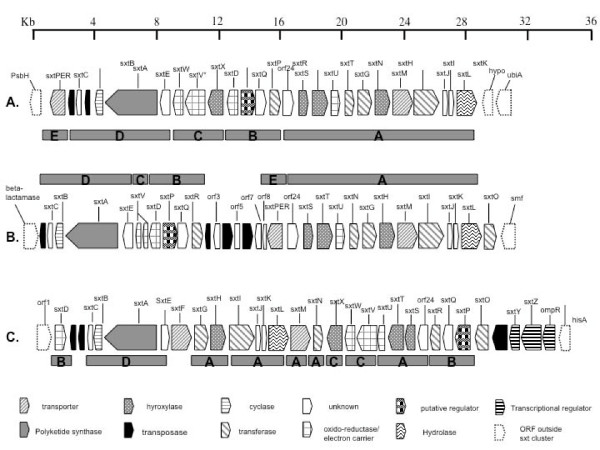
**Structure of the paralytic shellfish toxin biosynthesis cluster identified in A. *Aphanizomenon. sp*. NH-5, B. *Anabaena circinalis *AWQC131C, C. *Cylindrospermopsis raciborskii *T3**. The gene cluster schematic for *C. raciborskii *T3 has been adapted from kellmann *et al*. 2008 [29]. Segments A-E denote cluster fragments homologous in the three strains. The scale indicates length in thousand base pairs. ompR, transcriptional regulator of *ompR *family.

We have recently proposed a putative STX biosynthesis gene cluster in *Cylindrospermopsis raciborskii *T3 [29]. The putative STX biosynthesis gene cluster (*sxt*) in *C. raciborskii *T3 spans approximately 36 kb, and encodes genes involved in the biosynthesis, regulation and export of PSTs (Figure 2). Some of the genes identified in the *C. raciborskii *T3 gene cluster have not have been assigned a function due to their low level of structural homology to proteins in available databases. These genes may therefore represent novel enzyme families. Since *C. raciborskii *T3 is not genetically transformable, mutagenesis of the *sxt *cluster was not possible. The choice of identifying and characterizing the two novel putative PST biosynthesis gene clusters in *Anabaena *and *Aphanizomenon *was principally motivated by the detrimental effect the producer organisms have on water quality and public health. Furthermore, comparative analysis of the differences identified in toxin production, that is, the lack of neoSTX and all other N1-hydroxylated PSTs in *A. circinalis *AWQC131C, may provide further insights into the PST biosynthetic machinery [17,28]. The close taxonomic and phylogenetic relatedness of these two PST-producing organisms, also provides insights into the evolutionary history of the putative PST biosynthesis gene clusters, revealing whether they are of a common ancestor or laterally derived via horizontal gene transfer.

## Results and discussion

### Identification of PST gene clusters in *A. circinalis *AWQC131C and *Aph*. sp. NH-5

The gene cluster putatively responsible for the biosynthesis of PSTs in the cyanobacterium *C. raciborskii *T3 has recently been characterized by our group [29]. In an attempt to identify the putative PST biosynthesis gene cluster analogs, from the PST producing organisms *A. circinalis *AWQC131C and *Aph*. sp. NH-5, a reverse genetic approach was employed. The primer pair nodF and nodR (Table 1) was used in a degenerate PCR, targeting conserved regions in carbamoyltransferases (*sxtI*), which are putatively involved in the transfer of a carbamoyl group from carbamoyl phosphate onto the free hydroxymethyl side chain of the saxitoxin precursor [29,30]. A single amplicon of about 900 bp in size was amplified using genomic DNA isolated from both *A. circinalis *AWQC131C and *Aph*. sp. NH-5. The degenerate PCR products were then cloned and clone libraries were constructed, in an attempt to identify all the carbamoyltrasferases in these PST producing organisms. Screening of these clone libraries revealed only one gene fragment in each of the libraries. BLAST similarity searches showed homology between these gene fragments and other carbamoyltransferases. The *sxtI *gene homologs identified in *A. circinalis *AWQC131C and *Aph*. sp. NH-5 were 90% and 91% identical in sequence to the *sxtI *gene from *C. raciborskii *T3, respectively, and were 97% identical among themselves (Table 2) [29]. These gene fragments were consequently good candidates for *sxtI *homologs, and therefore also PST biosynthesis genes in these cyanobacteria.

**Table 1:** PCR primer sequences used in this study

Name	Primer sequence (5'-3')	Reference
NodF	ATGGGHYTRGCHCCHTAYGG	[29]
NodR	CCBCGYACRTTRAAKGABGTRTT	[29]
AnastartF	CGGGGGTATTTTTATTAGAC	This study
Ana30kbR	AGGGAATAGACACCGAAAGT	This study

**Table 2:** Similarity and predicted function of PST biosynthesis genes

Gene	A. circinalis AWQC131C gene size (bp)	A. circinalis AWQC131C identity to C. raciborskii T3	Aph. sp. NH-5 gene size bp	Aph. sp. NH-5 identity to C. raciborskii T3	Aph. sp. NH-5/A. circinalis AWQC131C identity	Closest BLAST match	Putative function
sxtC	285	90%	285	90%	99%	ABI75092.1 SxtC (354 bp)	Unknown
sxtB	978	87%	969	88%	97%	ABI75093.1 SxtB	Cyclisation
sxtA	3705	90%	3705	90%	99%	ABI75094.1 SxtA	Loading of ACP, methylation, ACP, Claisen condensation
sxtE	477	64%	363	83%	75%	ABI75095.1 SxtE	Unknown
sxtV	Disrupted	-----	1663	90%	-----	SxtV ABI75107.1	Inactive
sxtD	759	88%	759	89%	98%	SxtD ABI75089.1	Desaturation
sxtP	1449	53%	1443	58%	97%	SxtP ABI75114.1	Regulator/pilli formation
sxtQ	777	91%	777	90%	98%	SxtQ ABI75113.1	Unknown
sxtR	804	88%	777	87%	87%	SxtR ABI75112.1	Acyl-CoA N-acyltransferase
sxtPER	957	-----	1059	-----	82%	Nostoc punctiforme PCC 73102 ACC82015.1	Transporter
orf24	627	83%	576	92%	86%	Orf24 ABI75111.1	Unknown
sxtS	726	87%	729	75%	90%	sxtS ABI75110.1	Ring formation
sxtT	1020	82%	1020	86%	95%	sxtT ABI75109.1	C-12 hydroxylation
sxtU	750	88%	750	87%	97%	sxtU ABI75108.1	Reduction of C-1
sxtN	870	86%	846	89%	92%	sxtN ABI75104.1	Sulfotransfer
sxtG	1134	93%	1134	94%	98%	sxtG ACF94638.1	Amidinotransfer
sxtH	1020	84%	1020	87%	95%	sxtH ACF94646.1	C-12 hydroxylation
sxtM	1458	82%	1458	76%	92%	sxtM ABI75103.1	Export of PSTs
sxtI	1839	90%	1839	91%	97%	sxtI ABI75099.1	Carbamoylation
sxtJ	405	82%	405	82%	98%	sxtJ ABI75100.1	Unknown
sxtK	165	93%	165	90%	96%	sxtK ABI75101.1	Unknown
sxtL	1281	83%	1278	85%	92%	sxtL ABI75102.1	Decarbamoylation
sxtO	570	12%	-----	-----	-----	Cyanothece sp. PCC 8801 adenylylsulfate kinase ZP_02939438.1	PAPS biosynthesis
sxtW	-----	-----	327	95%	-----	sxtW ABI75106.1	Ferredoxin/electron carrier
sxtX	-----	-----	756	87%	-----	sxtX ABI75105.1	N-1 hydroxylation
orf3	302	-----	-----	-----	-----	sxtN ABI75104.1	Disrupted sulfotransferase
orf5	459	-----	-----	-----	-----	sxtN ABI75104.1	Disrupted sulfotransferase
orf7	226	-----	-----	-----	-----	sxtN ABI75104.1	Disrupted sulfotransferase
orf8	98	-----	-----	-----	-----	sxtW ABI75106.1	Disrupted ferredoxin

The saxitoxin biosynthesis genes in *Anabaena circinalis *AWQC131C and *Aphanizomenon sp*. NH-5, their similarities and bioinformatically-deduced functions. Closest BLAST match column refers to both *A. circinalis *AWQC131C &*Aph*. sp. NH-5, if present in both species.

In order to obtain the DNA sequences of the entire putative PST biosynthesis gene clusters in both *A. circinalis *AWQC131C and *Aph*. sp. NH-5, a gene walking technique (known as pan-handle PCR) was employed [31]. Following numerous rounds of sequencing-out and gene-walking reactions from the putative *sxtI *homologues, the entire gene cluster in both organisms was identified, sequenced and characterized (Figure 2). The putative PST gene cluster in both organisms is of comparable size. The *A. circinalis *AWQC131C *sxt *gene cluster spans 29 kb, flanked by a β-lactamase, a gene involved in antibiotic resistance, at the 5' end and a *smf *gene homolog, believed to be involved in DNA uptake at the 3' end (Figure 2). The *Aph*. sp. NH-5 *sxt *gene cluster is slightly smaller and covers 27.5 kb. It is flanked on both sides by genes coding for photosynthesis machinery, at the 5' end by *psbH*, a photosystem II reaction centre gene and at the 3' end by a prenyltransferase involved in the synthesis of ubiquinone (Figure 2).

### Characterisation of the PST biosynthesis gene clusters

The major PSTs identified in *Aph*. sp. NH-5 are neoSTX and STX [17] and other toxic fractions that were not identified, although the methods used were not highly sensitive. While the major PSTs identified in *A. circinalis *AWQC131C were STX, GTX2/3, C1/2, dcSTX and dcGTX2/3 [28]. This observed difference in the toxin profiles is most probably the result of the different genetic backgrounds in these producer organisms, as modification reactions to the STX parent molecule, carried out by the *sxt *gene cluster tailoring enzymes, putatively result in the formation of the various PSTs.

Both gene clusters characterized in this study contain the same set of genes identified in the putative PST biosynthesis gene cluster (*sxt*) of *C. raciborskii *T3 with some exceptions (Table 2) [29]. Specifically, they do not contain the genes *sxtY*, *sxtZ *and *ompR*, believed to be involved in signal transduction and the transcriptional regulation of PST production in *C. raciborskii *T3, and therefore might be regulated in a different manner, however it can not be completely excluded that these genes have diverged and transposed to a different locus in the genome. Furthermore, they do not contain the gene *sxtF *putatively involved in PST transport. Both the *A. circinalis *AWQC131C and the *Aph*. sp. NH-5 PST gene clusters contain a gene, denoted *sxtPER*. Curiously, *sxtPER*, a gene most similar to members of the drug and metabolite transport family, has not been identified in the *C. raciborskii *T3 PST gene cluster.*sxtPER *might therefore fulfil a similar role to *sxtF *in *C. raciborskii *T3 and is postulated to be involved in the transport of PSTs in *Anabaena *and *Aphanizomenon*. Interestingly, *sxtPER *has an evolutionary history apparently distinct from *sxtM *and *sxtF *and represents a different lineage, while *sxtF *seems to have arisen by a gene duplication event of *sxtM*. The phylogeny, function and distribution of cyanobacterial MATE genes are addressed in a separate publication (Pengelly JJL, Mihali TK, Neilan BA: Identification, phylogeny and expression of novel members of the multi-drug and toxic compound extrusion (MATE) family from cyanobacteria, submitted).

Furthermore, the *Aph*. sp. NH-5 *sxt *gene cluster is missing a *sxtO *homolog, an adenylylsulfate kinase. *sxtO *is present in the *A. circinalis *AWQC131C *sxt *gene cluster and the *C. raciborskii *T3 *sxt *gene cluster. *sxtO *is most similar to adenylylsulfate kinases that are involved in the formation of 5'-phosphoadenosine 3'-phosphosulfate (PAPS), which is the sulfate donor for PAPS dependant sulfotransferases (Table 2) [29]. The toxin profile of *Aph*. sp. NH-5 has not been fully characterized, however, it has been shown to produce STX and neoSTX and possibly other unidentified toxic fractions [17]. It is therefore not possible to asses whether this strain actually produces sulfated PSTs. As adenylylsulfate kinases are not unique to the PST gene clusters, and are ubiquitous enzymes needed for all PAPS dependant sulfotransferases, it seems plausible that this genetic mutation could be complemented by an additional adenylylsulfate kinase elsewhere in the genome of *Aph*. sp. NH-5. Curiously, the *sxtO *homolog identified in *A. circinalis *AWQC131C *sxt *gene cluster has very low similarity to *sxtO *identified in *C. raciborskii *T3 (Table 2), and is more similar to other cyanobacterial derived adenylylsulfate kinases. This observation further supports the idea that PST biosynthesis accessory genes may be complimented by gene homologs in the genome of the producer organism.

**Figure 3:**
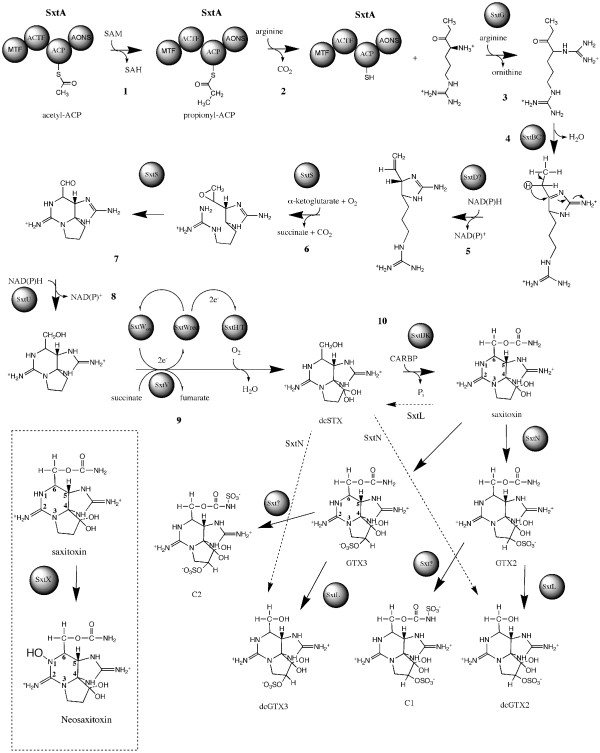
**Proposed saxitoxin biosynthetic pathway**. Dotted lines indicate additional but not essential tailoring reactions, dotted box indicates reaction only present in *Aph. sp*. NH-5. See text for detail. Adopted and modified from [29].

On the other hand, the *A. circinalis *AWQC131C putative PST biosynthesis gene cluster does not contain the tailoring gene *sxtX*, which is most similar to a cephalosporine hydroxylase, and presumably responsible for the hydroxylation of N-1 in STX, thereby converting STX to neoSTX (Figure 3). Hence, *sxtX *is putatively involved in the formation of all the analogs containing a hydroxyl at N-1 (Figure 1). In accordance with this finding, *A. circinalis *AWQC131C does not produce any STX congeners that contain a hydroxyl at N-1. In contrast the *Aph*. sp. NH-5 *sxt *gene cluster does contain the tailoring enzyme gene *sxtX*, and correspondingly been shown to produce the N-1 hydroxylated analog neoSTX [17], further affirming its putative role in the PST biosynthesis gene cluster.

The *A. circinalis *AWQC131C *sxt *gene cluster does not contain the gene *sxtW*, which is present in the *Aph*. sp. NH-5 and the *C. raciborskii *T3 *sxt *gene clusters. *sxtW *is most similar to a ferredoxin and is believed to be involved in electron transport, required for the hydroxylation of C-12 by the two ring-hydroxylating dioxygenases, encoded by *sxtT *and *sxtH*. Curiously, the *A. circinalis *AWQC131C *sxt *gene cluster contains a small (98 bp), disrupted ORF denoted *orf8*, with similarity to *sxtW *and other ferredoxin genes (Table 2). *Orf8 *might therefore represent yet another fragment of a PST biosynthesis accessory gene that has been inactivated. It is plausible that an endogenous ferredoxin complements for its loss in *A. circinalis *AWQC131C, since homologs of ferredoxin (Accession no. Npun_R5178, Ava_5008) have been detected in several recently released cyanobacterial genomes, as evident from BLAST homology searches.

*sxtV*, a gene most similar to succinate dehydrogenases, originally identified in the *C. raciborskii *T3 PST gene cluster, is also believed to be involved in this electron transport mechanism. Only a fragmented part of this gene is present in the *A. circinalis *AWQC131C *sxt *gene cluster, while *sxtV *in the *Aph*. sp. NH-5 *sxt *gene cluster contains a stop codon interrupting the ORF. It is therefore postulated that this gene is also complemented by another locus in the genome of these producer organisms or is not essential for PST biosynthesis.

Interestingly, the *A. circinalis *AWQC131C *sxt *gene cluster also contains partial transposase-like sequences, which interrupt a putative ORF and split it into 3 different fragmented partial ORFs, denoted *orf3*, *orf5 *and *orf7 *(Figure 2). This fragmented gene is partially similar to *sxtN*, homologs of which are present in the *A. circinalis *AWQC131C and *Aph*. sp. NH-5 *sxt *gene clusters, and putatively involved in the sulfation of STX and its derivative [29]. Due to its fragmentation and short coding sequence we were unable to assign a putative role for this gene, but it is assumed to be inactive.

### Structural organization

The organization of genes in the, *A. circinalis *AWQC131C and *Aph*. sp. NH-5 *sxt *gene clusters, is very conserved, and has less common features with the organization of the *C. raciborskii *T3 *sxt *cluster (Figure 2). As depicted in Figure 2, the putative PST biosynthesis gene cluster can be divided into five segments of coding DNA, denoted as A-E. The *A. circinalis *AWQC131C *sxt *gene cluster contains three truncated ORFs (*orf 3*, *5 *and *7*) intersected by transposases, however, these are absent from the *Aph*. sp. NH-5 *sxt *gene cluster. In the *Aph*. sp. NH-5 *sxt *gene cluster, segment C contains the genes *sxtX, sxtV *and *sxtW*, whereas in the *A. circinalis *AWQC131C *sxt *gene cluster most of segment C is missing, only a fragmented section of *sxtV *is retained. Furthermore, in the *A. circinalis *AWQC131C putative PST biosynthesis gene cluster, segment E is adjacent to segment A, whereas in *Aph*. sp. NH-5 segment E is connected to segment D (Figure 2). Therefore, it seems plausible to assume that the *A. circinalis *AWQC131C *sxt *gene cluster has had at least two DNA recombination events as compared to the *Aph*. sp. NH-5 *sxt *gene cluster, with one event causing the truncation of segment C, thereby losing the genes *sxtX, sxtV *and *sxtW*. The other recombination event would have involved segment E, which contains the gene denoted *sxtPER*, to relocate from its 5' position in *Aph*. sp. NH-5 to the central region of the gene cluster in *A. circinalis *AWQC131C (Figure 2).

### Biosynthesis of PST congeners

A detailed description of the proposed biosynthesis of the STX parent molecule has recently been published by our group [29]. Briefly, biosynthesis is initiated with SxtA, which contains 4 catalytic domains (Table 2, Figure 3 step 1–2). A methyltransferase domain (*sxtA1*), a GNAT domain (*sxtA2*) (loading of acyl carrier protein), an acyl carrier protein (ACP) domain (*sxtA3*) and an AONS domain (*sxtA4*) (which acts as a condensation domain). The predicted reaction sequence of SxtA, is the loading of the ACP (SxtA3) with acetate from acetyl-CoA by sxtA2, followed by the SxtA1-catalysed methylation of acetyl-ACP, converting it to propionyl-ACP. The class II aminotransferase domain (AONS), SxtA4, then performs a Claisen-condensation between propionyl-ACP and arginine. The product of SxtA is thus 4-amino-3-oxo-guanidinoheptane. The next step (Figure 3 step 3) is carried out by SxtG, an amidinotransferase that transfers an amidino group from arginine onto the product of SxtA, producing 4,7-diguanidino-3-oxoheptane. This compound is then condensed via SxtB, which is most similar to a cytidine deaminase, in a retro-aldol-like condensation (Figure 3 step 4) and the first heterocycle is formed. The following step (Figure 3 step 5) involves SxtD a desaturase, which introduces a double bond between C-1 and C-5 resulting in the 1,2-H shift between C-5 and C-6. SxtS a 2-oxoglutarate-dependent (2OG) dioxygenase, then performs the consecutive epoxidation of the new double bond, and opening of the epoxide to an aldehyde with concomitant bicyclisation (Figure 3 steps 6–7). The dehydrogenase SxtU then reduces the terminal aldehyde group of the STX precursor (Figure 3 step 8). Thereafter (Figure 3 step 9), SxtH and SxtT, each coding a terminal oxygenase subunit of bacterial phenyl-propionate and related ring-hydroxylating dioxygenases, catalyse the consecutive hydroxylation of C-12, forming dcSTX. Thereafter, SxtI in conjunction with SxtJ and SxtK catalyse a carbamoyltrasfer from carbamoylphosphate onto the free hydroxyl at C-13, forming saxitoxin (STX).

Subsequently, tailoring reactions catalyse the formation of the PST analogues, which are derivatives of the parent molecule STX. One of the most potent STX derivatives is the N-1 hydroxylated saxitoxin analogue neoSTX. As previously mentioned, SxtX has been predicted to be involved in the N-1 hydroxylation of saxitoxin [29]. Therefore we postulate that SxtX, which is not encoded in the *A. circinalis *AWQC131C PST biosynthesis gene cluster, carries out the conversion of STX to neoSTX (Figure 3). *A. circinalis *AWQC131C is known to produce N-21 and O-22 sulfated STX analogues (GTX2/3, C1/2, dcGTX2/3). The activity of two PAPS dependent sulfotransferases, which were specific for the N-21 of STX and GTX-3/2, and O-22 of 11-hydroxy STX, respectively, have been described from the PSP toxin-producing dinoflagellate *Gymnodinium catenatum*, although no protein sequence information was obtained due to instability of the enzyme and low yield [32,33]. Both putative PST biosynthesis gene clusters identified in this study contain a gene denoted *sxtN*, which is most similar to estrogen sulfotransferase and putatively responsible for the sulfation of STX. Therefore, the protein encoded by *sxtN*, is postulated to be responsible for the sulfation of STX at O-22 resulting in the formation of GTX2 and GTX3 (Figure 3). Alternatively SxtN could sulfate N-21 of STX resulting in the formation of C1 and C2. It is further possible that the enzyme catalyses both reactions, or that a sulfotransferase not encoded in the PST biosynthesis gene cluster is involved. Whether the product of this gene sulfates O-22 or N-21 of STX could not be determined at this point, and will require heterologous expression of the enzyme to determine its natural substrate specificities. dcSTX derivatives are postulated to result from the hydrolytic cleavage of carbamoylated STXs. The candidate enzyme encoded by *sxtL*, harbouring homology to GDSL-lipases, has been proposed to catalyse this cleavage [29]. SxtL would therefore catalyse the formation of dcGTX2/3 from GTX2/3, and putatively convert STX to dcSTX (Figure 3). The exact sequence of reactions is not clear at this point, as there is need for heterologous expression of each of these tailoring enzymes and their substrate specificities have to be confirmed experimentally.

### Phylogeny of the PST biosynthesis genes

**Figure 4:**
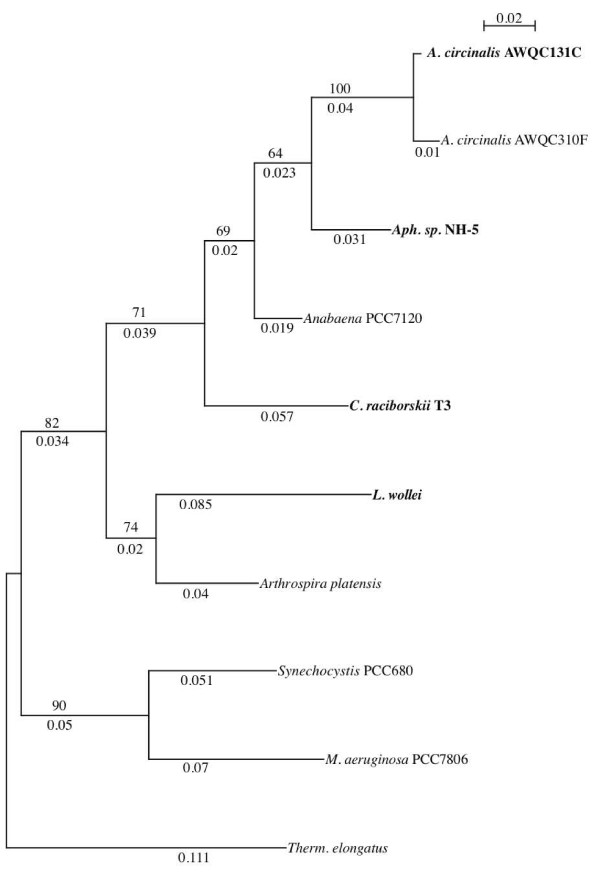
**Phylogenetic tree of the 685 bp partial 16S rRNA gene from saxitoxin-producing cyanobateria**. *Anabaena circinalis *AWQC131C and *Aphanizomenon sp*. NH-5 show highest similarity to each other compared to *C. raciborskii *T3 or *L. wollei*. Bootstrap confidence levels are indicated on top of each branch. Branch lengths are indicated below (nucleotide substitutions per 100 character positions). Trees were reconstructed using PhyML using a GTR+G model with 1000 bootstrap replicas. Bold type indicates known PST producers.

It is interesting to note that the organization of the putative PST gene clusters in *A. circinalis *AWQC131C and *Aph*. sp. NH-5 are more similar to each other, than to the organization in the recently identified putative PST gene cluster in *C. raciborskii *T3 (Figure 2). Furthermore, the *A. circinalis *AWQC131C and *Aph*. sp. NH-5 *sxt *genes show overall greater sequence similarity to each other than to the recently identified *sxt *genes in *C. raciborskii *T3 (Table 2). In an attempt to reconstruct the taxonomic phylogeny of the PST producing cyanobacteria *A. circinalis *AWQC131C, *Aph*. sp. NH-5, *C. raciborskii *T3 and *L. wollei*, a phylogenetic approach was used. 16S rRNA gene sequences for the PST-producing and non-producing cyanobacteria were retrieved from GenBank (Table 3). An alignment of these sequences was created and a phylogenetic tree based on a 685 bp segment of the 16S rRNA gene was reconstructed. As evident from the phylogenetic tree (Figure 4), *A. circinalis *AWQC131C and *Aph*. sp. NH-5 form a supported clade together with the (non-toxic) *A. circinalis *AWQC310F (64% bs) and are more closely related to each other than to *C. raciborskii *T3. Consequently, the topological organization of the PST biosynthesis gene cluster is reflected by the phylogeny of the producer organism, and they therefore most likely have a paralleled evolution.

**Table 3:** 16S rDNA sequences

Strain	Gene	Accession no.
Anabaena circinalis AWQC131C	16S rDNA	AF247589
Anabaena circinalis AWQC310F	16S rDNA	AF247579
Aphanizomenon sp. NH-5	16S rDNA	AF425995
Anabaena/Nostoc sp. PCC7120	16S rDNA	NC 003272
Cylindrospermopsis raciborskii T3	16S rDNA	EU439566
Lyngbya wollei	16S rDNA	EU603708
Arthrospira platensis	16S rDNA	EU427543
Synechocystis sp. PCC6803	16S rDNA	BA000022
Microcystis aeruginosa PCC7806	16S rDNA	MAU03402
Thermosynechococcus elongatus BP-1	16S rDNA	BA000039

16S rDNA sequences used in this study and their corresponding accession numbers.

**Figure 5:**
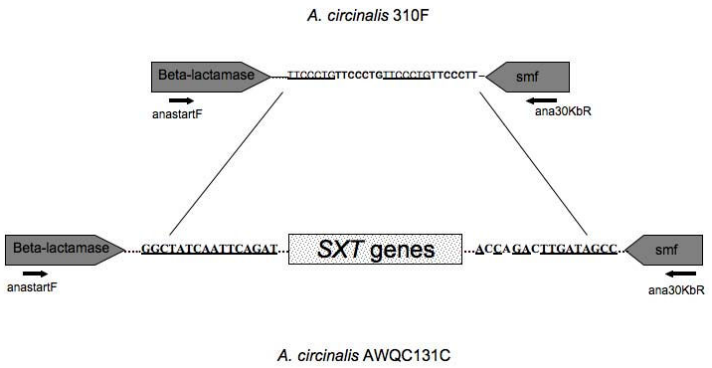
**Putative insertion/excision site of the paralytic shellfish toxin gene cluster, in Australian *Anabaena circinalis *strains**. Diagram not to scale. See text for details.

Australian isolates of *A. circinalis *have been shown to be phylogenetically related, forming two distinct branches [24]. Furthermore, genetic screening has shown that only toxic isolates contain the putative genes for PST biosynthesis [29]. An attempt was made to identify the insertion/excision point of the putative PST gene cluster in the Australian *A. circinalis *isolates. PCR primers AnastartF and Ana30kbR were designed based on the sequences obtained from *A. circinalis *AWQC131C (Table 1), as strain representative of the PST-producing lineage. These primers were designed within the ORF of the β-lactamase at the 5' end, and *smf *at the 3' end of the PST gene cluster in *A. circinalis *AWQC131C (Figure 5). A PCR reaction with this primer set was performed using genomic DNA isolated from the non-toxic *A. circinalis *310F strain belonging to the non-PST producing clade, and the PST-producing *A. circinalis *AWQC131C. The PCR resulted in the amplification of a 1.6 kb segment of DNA from *A. circinalis *310F, whereas genomic DNA from *A. circinalis *AWQC131C did not yield a product. The predicted distance between the primer set in *A. circinalis *AWQC131C genomic DNA was over 29 Kb and therefore an amplicon was not expected using the conditions described. Sequencing followed by bioinformatic analysis of the 1.6 kb amplicon from *A. circinalis *310F revealed two ORFs, one at the 5' end that was similar to a β-lactamase, and another at the 3' end similar to a *smf *gene, both homologous to the genes identified on either ends of the putative PST biosynthesis gene cluster in *A. circinalis *AWQC131C. A closer analysis of the *A. circinalis *310F amplified region revealed the presence of four direct repeats of the sequence **TTCCCTG **within the intragenic spacer identified between the two flanking ORFs (Figure 5). Insertion sites which are targeted by a transposon are usually innocuous and do not contain repeats, however, the presence of direct repeats is indicative of an excision event that has followed the insertion of a transposon [34]. Further examination of the non-coding regions flanking the PST biosynthesis gene cluster in *A. circinalis *AWQC131C revealed a relic inverted repeat of the sequence **GGCTATCAATTCAGAT **on either side of the PST biosynthesis gene cluster that could also indicate a past transposition event. The combination of these findings supports the theory that the non-toxic *A. circinalis *strains have lost the putative PST biosynthesis gene cluster during an excision event. Together with the observation that the organization of the putative PST biosynthesis gene clusters resembles the phylogeny of the producer organisms, as well as the fact that *A. circinalis *AWQC131C and *Aph*. sp. NH-5 PST genes are overall more similar to each other than to the *C. raciborskii *T3 PST genes (Table 2), might imply that the *sxt *gene cluster is of ancient origin. Therefore, the sporadic distribution of PST biosynthesis throughout phylogeny could be explained by the loss of function in non-toxic strains, rather than the acquisition of one or more genes during more recent horizontal transfer in PST producing toxic strains. A similar evolutionary history has recently been proposed for the sporadic distribution of another cyanobacterial toxin biosynthesis gene cluster encoding microcystin synthetase [35].

## Conclusion

Here we have described the identification, annotation and characterisation of the putative PST biosynthesis gene clusters in *A. circinalis *AWQC131C and *Aph. sp*. NH-5. The putative PST biosynthesis gene cluster presents a mosaic structure, whereby genes have apparently transposed in segments of varying size, resulting in different gene arrangements in all three *sxt *clusters sequenced so far. This gene cluster arrangement is in agreement with a mobile genetic element, further supported by the identification of multiple transposase like sequences in other recently characterized cyanobacterial toxin and secondary metabolite gene clusters [31,36,37]. The putative point of insertion/excision of the *sxt *gene cluster in Australian isolates of *A. circinalis *was also identified. The gene cluster organizational structure and gene sequence identity seem to reflect the phylogeny of the producer organisms, indicating that the gene clusters might have an ancient origin, or that their lateral transfer was also an ancient event. The further sequencing and characterisation of cyanobacterial PST biosynthesis gene clusters in different producer organisms, with different toxin profiles, may provide new insights into the biosynthetic machinery and the enzymes involved at each step. The availability of more putative PST biosynthesis gene sequences from different producer organisms will also enable the better monitoring of algal blooms by water authorities, including the use of PCR-based early warning systems. Future studies into the transcriptional control and physiological roles of the PSTs, furthering our ability to predict and prevent the formation of harmful algal blooms, is also possible given these gene sequences and associated regulatory regions. The knowledge we gain from the characterisation of the PST biosynthesis gene clusters, including the identity and sequence of the genes involved in the biosynthesis, may also afford the identification of these gene cluster in dinoflagellates, the cause of human mortalities and significant financial loss to the tourism and shellfish industries.

## Methods

### Cyanobacterial strains

*A. circinalis *AWQC131C and *Anabaena circinalis *310F [24] were grown in Jaworski medium [38] in static batch culture at 26°C under continuous illumination (10 μmol m^-2^s^-1^), *Aph. sp*. NH-5 and *L. wollei *freeze-dried cultures were kindly supplied by W. W. Carmichael [17,19].

### DNA extraction

Total genomic DNA was extracted from cyanobacterial cells using the Mo Bio PowerPlant DNA isolation kit (Carlsbad, CA) in accordance with the manufacturers instructions. Genomic DNA was stored at -20°C.

### PCR

Insertion/excision point PCR was carried out using the primer set AnastartF and Ana30kbR (Table 1), and performed in 20 μl reaction volumes containing 1 × *Taq *polymerase buffer, 2.5 mM MgCl_2_, 0.2 mM deoxynucleotide triphosphates, 10 pmol each of the forward and reverse primers, 50 ng genomic DNA and 0.2 U of *Taq *polymerase (Fisher Biotech, Australia). Thermal cycling was performed in a GeneAmp PCR System 2400 Thermal cycler (Perkin Elmer Corporation, Norwalk, CT). Cycling began with a denaturing step at 94°C for 4 min followed by 30 cycles of denaturation at 94°C for 10 s, primer annealing at 55°C for 25 s and a DNA strand extension at 72°C for 2 min. Amplification was completed by a final extension step at 72°C for 7 min.

### Degenerate PCR

Degenerate PCR primers targeting the carbamoyltransferase *sxtI *used in this study, nodF and nodR (Table 1), were designed *ex-silico *from sequence alignments using ClustalX [39]. Degenerate PCR was performed in 20 μl reaction volumes containing 1 × *Taq *polymerase buffer, 2.5 mM MgCl_2_, 0.2 mM deoxynucleotide triphosphates, 25 pmol each of the forward and reverse primers, 50 ng of genomic DNA and 0.2 U of *Taq *polymerase (Fisher Biotech, Australia). Thermal cycling was performed in a GeneAmp PCR System 2400 Thermal cycler (Perkin Elmer Corporation, Norwalk, CT). Cycling began with a denaturing step at 94°C for 4 min followed by 35 cycles of denaturation at 94°C for 10 s, primer annealing at 50°C for 30 s and a DNA strand extension at 72°C for 45 s. Amplification was completed by a final extension step at 72°C for 5 min.

### Gene walking

The sequence of unknown regions that were flanking candidate genes was determined by an adaptor-mediated (Pan-handle) PCR method [40] that was modified as previously described [31]. Short adaptor DNA was ligated to digested genomic DNA, and a specific genomic outward-facing primer in combination with an adaptor primer was then used to amplify the unknown regions of the genome. Twenty picomoles of T7 adaptor were added to each reaction mixture, containing 1 μg of genomic DNA, 10 U of blunt-ended restriction enzyme, and 5 U of T4 ligase (Promega) in 1 × One Phor All buffer (Amersham/Pharmacia). The one-step digestion and ligation reaction mixture was incubated at room temperature overnight. The single-stranded end of the adaptor was blocked in a solution containing 1 × PCR buffer (Fischer Biotech), 4 mM MgCl_2_, and 12.5 μM ddNTP with 1 U of *Taq *DNA polymerase (Fischer Biotech). Thermal cycling was performed in a PCR Sprint temperature cycling system machine (Hybaid Ltd) with an initial step at 70°C for 15 min followed by 10 cycles of DNA denaturation at 95°C for 10 s, DNA reannealing at 40°C for 1 min, and extension of the strand with ddNTP at 70°C for 1 min. Following the PCR cycles, the reaction mixture was incubated with 1 U of shrimp alkaline phosphatase (Boehringer Mannheim, Göttingen, Germany) at 37°C for 20 min, and the enzyme was heat inactivated at 85°C for 5 min.

The flanking region PCR mixture contained 1 to 2 μl of adaptor-ligated DNA, 10 pmol of adaptor primer, 10 pmol of a genome-specific oligonucleotide primer and 0.5 U of a mixture of 10:1 *Taq *polymerase/PFU (Fischer Biotech, Australia). PCR cycling was performed as described above, with DNA strand extension at 72°C for 3 min. In addition, the primer annealing temperature was decreased for the first 10 cycles, by 1°C at each cycle, from 65 to 55°C, followed by primer annealing at 55°C for a further 25 cycles.

### Agarose gel electrophoresis

Amplified DNA was separated by gel electrophoresis with 1–2% agarose in TAE buffer (40 mM Tris-acetate, 1 mM EDTA, pH 7.8), and visualized by UV transillumination after staining with ethidium bromide (0.5 μg/ml). Where multiple amplicons were detected during the gel electrophoresis, single amplicons were excised from the gels and purified using the Promega Wizard^® ^SV Gel and PCR Clean-Up (Wisconsin, USA), prior to sequencing.

### Gene cloning

Clone libraries were created using the pGEM^®^-T Easy cloning kit from Promega (Wisconsin, USA) in accordance with the manufacturers instructions.

### DNA sequencing

Automated DNA sequencing was performed using the PRISM Big Dye cycle sequencing system and a model 373 sequencer (Applied Biosystems, Foster City, CA).

### Bioinformatic analysis

Sequence data were analysed using ABI Prism-Autoassembler software, while identity/similarity values to other translated sequences were determined using BLAST against the non-redundant (nr) data set, in conjunction with the National Center for Biotechnology Information (NIH, Bethesda, MD). Fugue blast  was used to identify distant homologs via sequence-structure comparisons. The gene clusters were assembled using the software package Phred, Phrap, and Consed , and open reading frames were identified manually.

### Phylogenetic analysis

16S rDNA sequences of the studied cyanobacteria were retrieved from GenBank (Table 3). Sequence alignments were preformed using ClustalX [41] and were manually edited to exclude ambiguous regions. Maximum-likelihood phylogenetic trees were reconstructed using PhyML, with four gamma substitution rate categories and a 1000 bootstrap replicas [42] (available on-line at ). The optimal DNA substitution model for the data set (GTR+G) was identified using the AiC in the program Modeltest [43].

### Nucleotide sequence accession number

Nucleotide sequences were submitted to GenBank. *A. circinalis *AWQC131C PST biosynthesis gene cluster is available under accession number DQ787201. *Aph*. sp. NH-5 PST biosynthesis gene cluster is available under accession number EU603710. *A. circinalis *310F insertion excision sequence is available under accession number EU603709.

## Abbreviations

PKS: polyketide synthase; PST: paralytic shellfish toxin; PSP: paralytic shellfish poisoning; STX: saxitoxin; GTX: gonyautoxin; dcSTX: decarbamoyl saxitoxin; neoSTX: neosaxitoxin; ACP: acyl carrier protein; PAPS: 5'-phosphoadenosine 3'-phosphosulfate.

## Authors' contributions

TKM carried out all experimental work, acquired, analysed and interpreted data and drafted the manuscript. RK participated in drafting of the manuscript and data interpretation. BAN participated in drafting of the manuscript, experimental design, data interpretation and supervised the overall progress of this project.
